# A parthenogenetic maternal and double paternal contribution to an ovotesticular disorder of sex development

**DOI:** 10.1186/1755-8166-7-16

**Published:** 2014-02-28

**Authors:** Xin-Yi Xia, Wei-Ping Wang, Tian-Fu Li, Wei-Wei Li, Qiu-Yue Wu, Na Li, Cui Zhang, Hong-Liu Gao, Xiao-Jun Li, Ying-Xia Cui

**Affiliations:** 1 Institute of Laboratory Medicine, Jinling Hospital, Nanjing University School of Medicine, Nanjing 210002, PR China

**Keywords:** Ovotesticular disorder of sex development, Parthenogenetic chimera, Molecular genetics

## Abstract

**Background:**

An ovotesticular disorder of sex development (OT-DSD) was rarely found in human. The mechanism causing such condition is poorly understood. We hereby reported a 11-year-old child with OT-DSD and a karyotype 46,XX/46,XY, a single maternal and double paternal genetic contribution to the patient.

**Results:**

Fluorescence *in situ* hybridization (FISH), blood grouping, HLA (human leukocyte antigen) haplotyping and a genome-wide scanning of lymphocytes with 398 short tandem repeat microsatellite markers were performed to investigate the origin of the cell lines concerned. ABO typing revealed that two populations of red cells were in the patient, which were group A and group B, both from paternal alleles. HLA haplotyping showed the patient had three haplotypes. Haplotype 1 was inherited from maternity, haplotype 2 and 3 were from paternity. The STR microsatellite analysis showed 25 of the 74 fully informative markers in both parents, three alleles were inherited: one of them was from mother, another two were from father. Seventeen of the thirty-eight paternal markers, the patient inherited two paternal alleles. For 121 informative maternal markers, the patient had a single maternal allele. There were two distinct alleles in locus DXS6810 and DXS1073 on X-chromosome, in which one was from the mother and the other from the father.

**Conclusions:**

The patient was a single maternal and double paternal genetic, which was a type of a parthenogenetic division of a maternal haploid nucleus into two identical nuclei, followed by fertilization by two spermatozoa and fusion of the two zygotes into a single individual at the early embryonic stage. To the best of our knowledge, this is the oldest OT-DSD case of parthenogenetic chimerism. These data provide additional evidence that a parthenogenetic maternal and double paternal contribution causes 46,XX/46,XY OT-DSD.

## Background

Ovotesticular disorder of sex development (OT-DSD), replaced the terminology ‘true hermaphrodite’ in 2006, is a rare condition of sexual differentiation and defined as the presence of ovarian and testicular tissue in the same individual [1,2]. It constitutes 3%-10% of the total DSD, and presents significant diagnostic and therapeutic challenges [3]. Few cases of OT-DSD with such studies have been reported [4-7]. The mechanism causing such 46,XX/46,XY chimerism is poorly understood [8]. We hereby report an 11-year–old child with an OT-DSD and a 46,XX/46,XY karyotype in the cultures from peripheral lymphocytes, skin fibroblasts and two kinds of gonadal tissues.

## Case presentation

**Figure 1:**
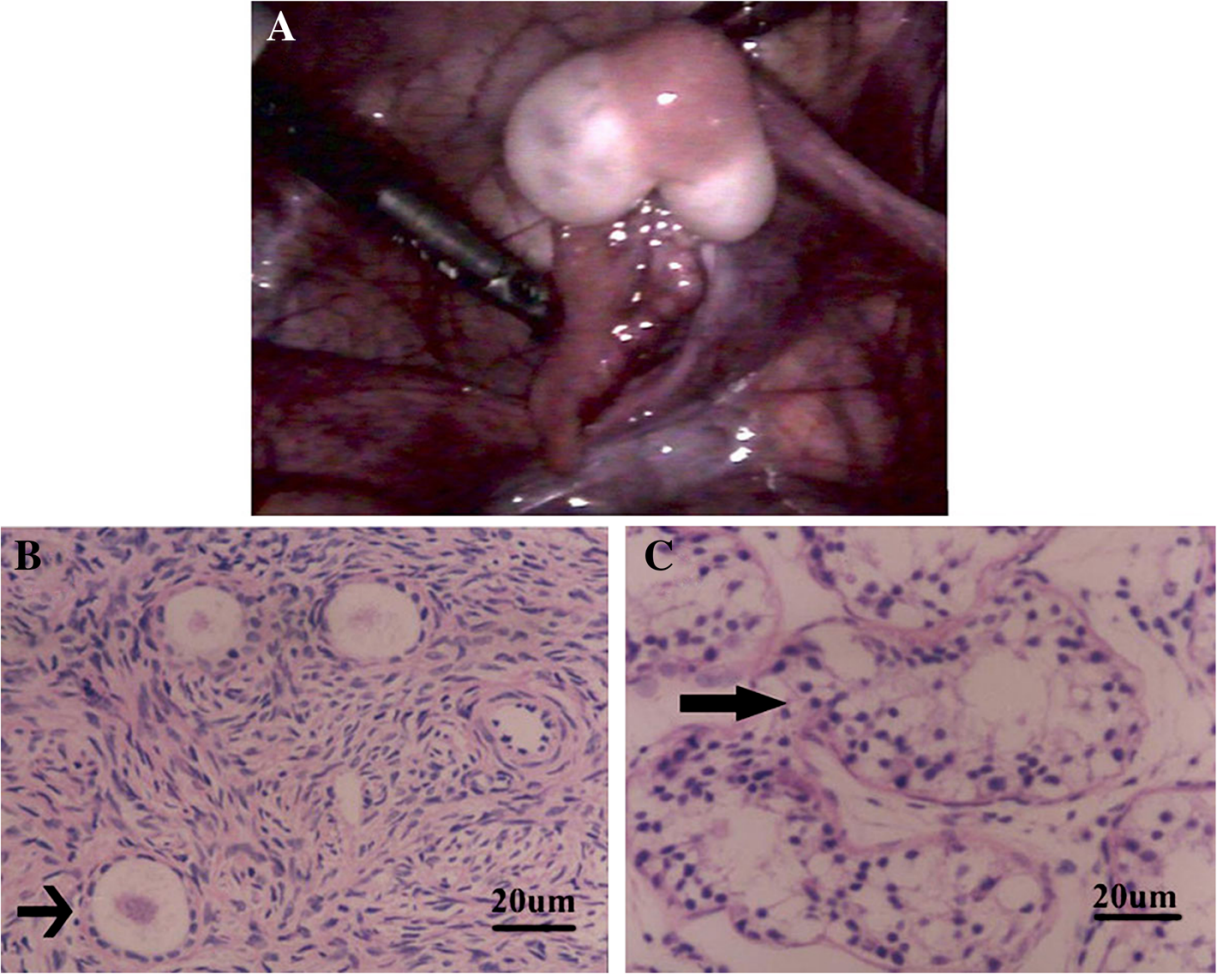
**Pathological analysis. (A)**: An ovotestis with a Fallopian tube. **(B)**: Ovarian tissue from the middle position of an ovotestis demonstrated by pathological analysis. Ovarian foci contain oocytes (arrow) in primary and tertiaryfollicles (HE staining). **(C)**: Testicular tissue from the both polars of an ovotestis demonstrated by pathological analysis. Arrow indicates seminiferous tubules (HE staining).

A 2-month-old infant was referred to our center because of ambiguous genitalia. The initial physiological examination found the infant had a 1.0 cm phallus, but both testes were unpalpable. The urethral meatus was located between the phallus and the vaginal orifice. A 46, XY karyotype was found at that moment. When the patient was 11 years old, a re-examination showed that the breasts developed symmetrically and normally as female at the age. The phallus length was 2.0-2.5 cm in drooping and 4.5-5.5 cm during erection, but both testes were still not palpable. In other respects the patient was developing as normal girls. The blood concentrations of gonadotrophins, prolactin, testosterone and progesterone were within the normal range for female children (23 IU/L, 75 mIU/L, 0.6 nmol/L and 6 IU/L). Normal uterus and Fallopian tube were found at laparoscopy. Bilateral gonads demonstrated ovotestis (Figure 1A). There were two kinds of tissue found at the position of both ovaries: a yellow tissue in the middle and white tissues at both poles. The white tissues showed ovarian structure (Figure 1B) and the yellow tissue, testicular structure (Figure 1C) through histopathological examination. Both the epididymides and vasa deferentia were also absent. During the follow-up visiting, the patient told us she had first menstruation at 11 years old and six months after her menstrual cycle became regular about 28 days long.

## Materials and methods

### Cytogenetic analysis and fluorescent *in situ* hybridization

Chromosomes were prepared from phytohemagglutinin-stimulated lymphocytes and cultured fibroblasts, from the patient’s dermatic, ovarian and testicular tissues, respectively. G-banding was according to standard techniques. Fifty G-banding metaphases were analyzed for each sample. Fluorescence *in situ* hybridization (FISH) using a mixture of probes specific for DXZ1 and DYZ3 (CEPX Spectrum green, CEPY Spectrum orange; Vysis, Downers Grove, IL) to determine X/Y-chromosome. FISH was performed on 500 metaphases for each sample from the patient. Tests were performed according to the manufacturer’s instructed protocols. Signals were visualized under an Olympus BX51 microscope (Center Valley, Pennsylvania) equipped with a cooled, charged coupled device camera and Cytovision 3.0 image analysis software (Applied Imaging, Sunderland, United Kingdom).

### Short tandem-repeat (STR) microsatellite markers

DNA was isolated from the peripheral blood of the patient and the parents. Using two commercial kits (PRISM Human Linkage Mapping Set v2.5, ABI, USA and PowerPlex 16, Promega, USA) with a total of 379 short- tandem-repeat (STR) microsatellite markers distributed over all 22 autosomes and 19 markers over X-chromosome. PCR products were analyzed in the ABI 377 DNA Sequencer (Applied Biosystems, USA). The results were analyzed by GeneMapper Software v4.0 (ABI).

### Blood grouping and HLA studies

Red cell typing for ABO (DiaMed-ID micro typing system, DiaMed, Switzerland) [9] and other blood-group antigens for the patient and the parents were assessed. Genomic samples of blood from the patient and the parents were also used for molecular typing of HLA class I and II markers by polymerase chain reaction (ABDR003vl-20020412 kits, Pel-Freez Clinical Systems, USA) sequence-specific primer amplification [10].

## Results

**Figure 2:**
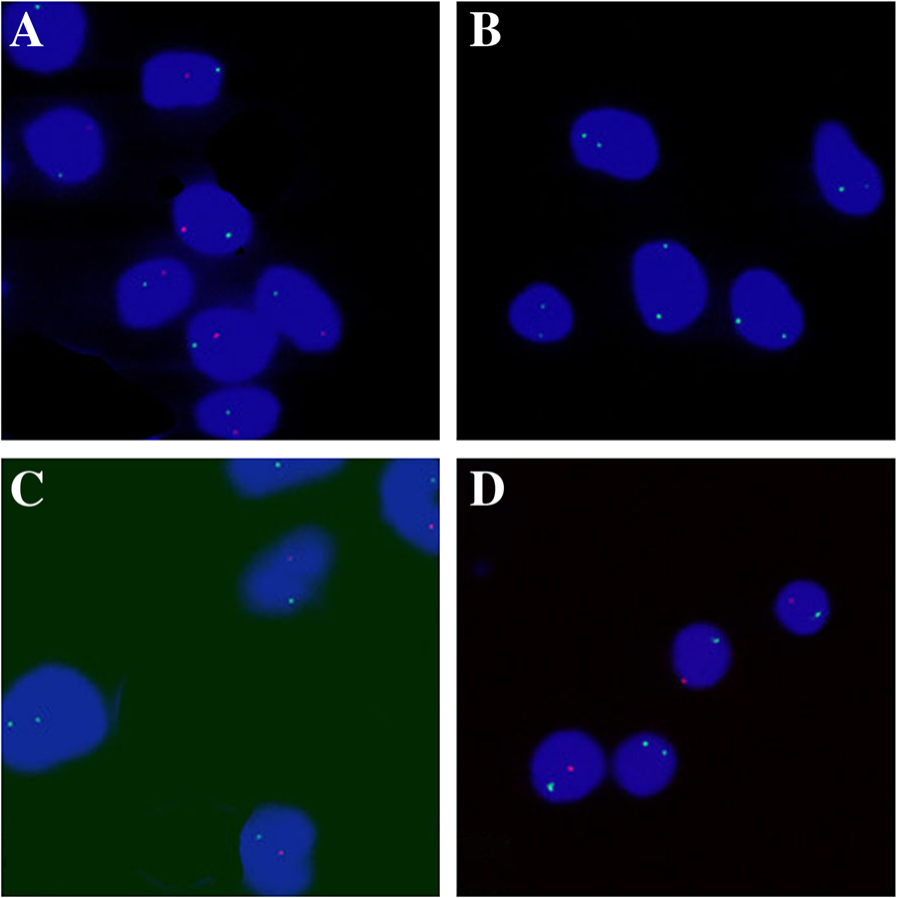
**FISH analysis. (A)**: FISH showed the predomination of XY cells over the interphase nuclei of the cultured-fibroblasts from the testicular tissues of the patient. **(B)**: FISH showed the predomination of XX cells over the interphase nuclei of the cultured-fibroblasts from the ovarian tissue of the patient. **(C)**: FISH showed the predomination XY cells interphase nuclei of the cultured-fibroblasts from the patient’s skin. **(D)**: FISH showed the predomination of XY cells over the interphase nuclei of the lymphocytes from the patient’s blood.

Chromosome analysis on peripheral lymphocyte and fibroblasts from the cultures of testicular tissue (Figure 2A), ovarian tissue (Figure 2B), skin tissue (Figure 2C) and patient’s blood (Figure 2D) revealed 46,XX/46,XY karyotype ratios of 23:27, 13:37, 44:6 and 5:45 (in 50 metaphases), respectively. The results of FISH were consistent with those of the cytogenetic analysis. The STR microsatellite analysis showed 25 of the 74 fully informative markers in both parents, the patient inherited three alleles, both paternal alleles and a single maternal allele and they distributed over 14 autosomes. For all 42 markers, 17 of the 38 only informative markers in the father, the patient showed three alleles and two of them originating from the father. For a total of 146 markers, 25 fully informative markers and 121 informative maternal markers, the patient was inherited a single maternal allele. For 4 X-chromosomal fully informative markers, the patient was showed two distinct alleles, in which one from the mother and the other from the father. None of 379 loci in autosomes showed 4 alleles. The ABO typing revealed that there were two populations of red cells in the patient, group A and group B. The mother was group O and the father group AB. The other blood groups the patient had such as Kidd, P, MNSs and Rh were the same as those of the parents. HLA haplotyping showed that the patient had three haplotypes, haplotype 1 was inherited from maternity, haplotype 2 and haplotype 3 from paternity. All details were shown in Table 1.

**Table 1:** Results of HLA haplotyping on the patient and the parents

	**Haplotype 1**	**Haplotype 2**	**Haplotype 3**
Mother	HLA-A*02, B*51, DRB1*09	HLA-A*0203, B*38, DRB1*16	
Father		HLA-A*24, B*46, DRB1*04	HLA-A*24, B*54, DRB1*09
Patient	HLA-A*02, B*51, DRB1*09	HLA-A*24, B*46, DRB1*04	HLA-A*24, B*54, DRB1*09

*Indicates an allele.

## Discussion

**Figure 3:**
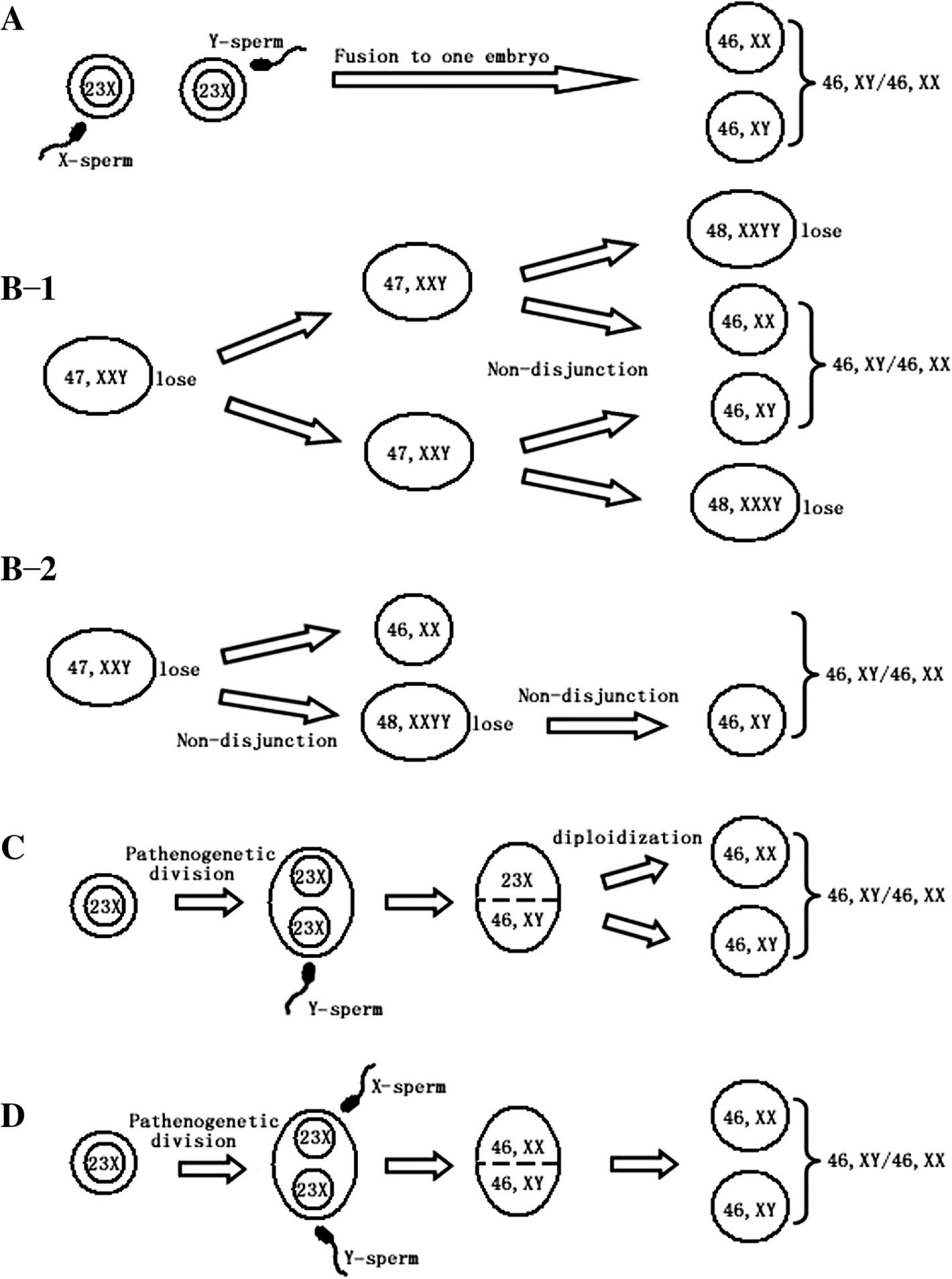
**Proposed mechanisms to explain chimera. (A)**: Fusion of two distinct embryos. **(B-1, B-2)**: Double mitotic nondisjunction of a 47,XXY zygote or sequential nondisjunction events in a 47,XXY single lineage. **(C)**: Fertilization of one parthenogenetic ovum by a spermatozoon, and the diploidization of the other. **(D)**: Parthenogenetic division of two identical ova, which were fertilized by two spermatozoa respectively.

We presented the clinical, cytogenetic, and molecular genetic findings of an OT-DSD of parthenogenetic chimera, to possess a single maternal genetic contribution and two paternal genetic contributions. As to the origin of a 46,XX/46,XY individual, four mechanisms have been identified by analysis of polymorphic microsatellite marker loci: (1) fusion of two distinct embryos [11], such a chimera showed two paternal and two maternal haploid genomes (Figure 3A). (2) A 47,XXY zygote during early embryonic development involved two separate non-disjunction events, resulting in the loss of X and Y, respectively [12]. Such a mosaic revealed a 46,XX/46,XY and a paternal and a maternal haploid genome, but two distinct alleles on X-chromosomal marker locus were identified, which implied that the two X-chromosomes in patient’s karyotype originated differently (Figure 3B-1 and B-2). (3) Fertilization of one parthenogenetic ovum by a spermatozoon and was diploidization of the other [13] (Figure 3C). Such a chimera also presented a paternal and a maternal haploid genome. This mechanism can be distinguished from the second by comparison of X-chromosomal marker locus and such a chimera had a single allele, which suggested the two X-chromosomes in patient’s karyotype originated identically. (4) A single haploid ovum divided parthenogenetically into two haploid ova, followed by double fertilization and fusion of the two zygotes into a single individual [14]. Such a chimera identified one single maternal allele and two paternal alleles (Figure 3D). The parthenogenetic phenomenon was also found in the IVF (in-vitro fertilization) laboratory [15] and induced by exposing tubal ova to high or low temperature, to a hypertonic or hypotonic solution or to stimulation with an electric current or to ether anesthesia.

Although no case of parthenogenesis has been reported in humans, it is possible that some individuals are partly parthenogenetic. For the first time, Strain *et al.*[15] described a ‘parthenogenetic chimera’ where one of the clones contained cells derived exclusively from a single duplicated maternal genome. Molecular studies using microsatellite markers showed a double paternal and a single maternal allelic contribution. In 1998, Giltay *et al.*[7] reported another patient with ambiguous genitalia. A parthenogenetic activation of the oocyte taking place before fertilization was suggested. In 2005, Chen *et al.*[16] presented the prenatal diagnosis, sonographic findings and, molecular genetic analysis of a 46,XX/46,XY true hermaphrodite chimera. Informative sex chromosome and pericentromeric autosome markers demonstrated double paternal and single maternal genetic contributions. In 2007, Souter *et al.*[10] reported a rare case of 46,XX/46,XY twins, twin A presented with ambiguous genitalia and twin B was a phenotypically normal male. The twins are chimeric and share a single genetic contribution from their mother but have two genetic contributions from their father thus supporting the existence of a third, previously unreported type of twinning. In 2008, Hersmus *et al.*[17] experienced two late-diagnosed children who presented with proximal hypospadias and bilateral scrotal gonads. One should consider the possibility of ovotesticular DSD when managing patients with proximal hypospadias even if both gonads are palpable in the scrotum.

The present case demonstrated that only a single maternal genetic contributes to the patient’s genome. First, an allele revealed in locus DXS6810 and locus DXS1073, which implied only a maternal X chromosomal contribution to the patient’s genome. Second, HLA haplotyping presented a single maternal allele and third, 146 marker loci on autosomes clearly demonstrated that the patient was inherited one maternal allele. These results are compatible with the involvement of one maternal gamete. With respect to the paternal genetic contribution, there were a number of evidences to identify two different spermatozoa from the father. For example, both a paternal X chromosome and a Y chromosome contributed to the patient karyotype; the ABO typing revealed two populations of red cells, group A and group B, both from paternal alleles; HLA haplotyping and 42 informative marker loci demonstrated the inheritance of the two paternal alleles, so the possibility of the first three mechanisms can be ruled out. It is very likely that in the present case there may be a parthenogenetic division of a haploid nucleus to give two identical nuclei followed by fertilization by two spermatozoa, just as the mode of origin of the chimerism described by Giltay *et al*. [7]. To the best of our knowledge, this is the oldest OT-DSD case of parthenogenetic chimerism.

## Conclusion

We presented the clinical, cytogenetic, and molecular genetic findings of an OT-DSD of parthenogenetic chimera, to possess a single maternal genetic contribution and two paternal genetic contributions. These data provide additional evidence that a parthenogenetic maternal and double paternal contribution causes 46,XX/46,XY OT-DSD. The mechanism causing such parthenogenetic chimera need to be further investigated.

## Consent

Written informed consent was obtained from the parents of the patient for publication of this Case report and any accompanying images. A copy of the written consent is available for review by the Editor-in-Chief of this journal.

## Abbreviations

OT-DSD: Ovotesticular disorder of sex development; FISH: Fluorescence *in situ* hybridization; HLA: Human leukocyte antigen; STR: Short tandem-repeat; IVF: In-vitro fertilization.

## Competing interest

The authors declare that they have no competing interests.

## Authors’ contributions

YXC, XJL, XYX, WPW, QYW, NL, CZ, HLG have made substantial contributions to conception and design, acquisition of data, analysis and interpretation of data. XYX, TFL, WWL, YXC have been involved in drafting the manuscript and revising it critically for important intellectual content. All authors read and approved the final manuscript.
